# Genetic basis of sorghum leaf width and its potential as a surrogate for transpiration efficiency

**DOI:** 10.1007/s00122-022-04167-z

**Published:** 2022-08-07

**Authors:** Xiaoyu Zhi, Graeme Hammer, Andrew Borrell, Yongfu Tao, Alex Wu, Colleen Hunt, Erik van Oosterom, Sean Reynolds Massey-Reed, Alan Cruickshank, Andries B. Potgieter, David Jordan, Emma Mace, Barbara George-Jaeggli

**Affiliations:** 1 Queensland Alliance for Agriculture and Food Innovation (QAAFI), Centre for Crop Science, The University of Queensland, Warwick, QLD Australia; 2 Queensland Alliance for Agriculture and Food Innovation (QAAFI), Centre for Crop Science, The University of Queensland, St Lucia, QLD Australia; 3 Queensland Alliance for Agriculture and Food Innovation (QAAFI), Centre for Crop Science, The University of Queensland, Gatton, QLD Australia; 4 Department of Agriculture and Fisheries (DAF), Hermitage Research Facility, Agri-Science Queensland, Warwick, QLD Australia; 5 Zhengzhou Research Base, State Key Laboratory of Cotton Biology, Institute of Cotton Research of Chinese Academy of Agricultural Sciences, Henan, China

## Abstract

**Key message:**

Leaf width was correlated with plant-level transpiration efficiency and associated with 19 QTL in sorghum, suggesting it could be a surrogate for transpiration efficiency in large breeding program.

**Abstract:**

Enhancing plant transpiration efficiency (TE) by reducing transpiration without compromising photosynthesis and yield is a desirable selection target in crop improvement programs. While narrow individual leaf width has been correlated with greater intrinsic water use efficiency in C_4_ species, the extent to which this translates to greater plant TE has not been investigated. The aims of this study were to evaluate the correlation of leaf width with TE at the whole-plant scale and investigate the genetic control of leaf width in sorghum. Two lysimetry experiments using 16 genotypes varying for stomatal conductance and three field trials using a large sorghum diversity panel (*n* = 701 lines) were conducted. Negative associations of leaf width with plant TE were found in the lysimetry experiments, suggesting narrow leaves may result in reduced plant transpiration without trade-offs in biomass accumulation. A wide range in width of the largest leaf was found in the sorghum diversity panel with consistent ranking among sorghum races, suggesting that environmental adaptation may have a role in modifying leaf width. Nineteen QTL were identified by genome-wide association studies on leaf width adjusted for flowering time. The QTL identified showed high levels of correspondence with those in maize and rice, suggesting similarities in the genetic control of leaf width across cereals. Three a priori candidate genes for leaf width, previously found to regulate dorsoventrality, were identified based on a 1-cM threshold. This study provides useful physiological and genetic insights for potential manipulation of leaf width to improve plant adaptation to diverse environments.

**Supplementary Information:**

The online version contains supplementary material available at 10.1007/s00122-022-04167-z.

## Introduction

Leaf size is an important determinant of canopy leaf area development, thus influencing crop photosynthesis, transpiration and yield (Duncan et al. 1967; Kholová et al. 2014; Zhu et al. 2012). In sorghum (*Sorghum bicolor* (L.) Moench) and maize (*Zea mays* L.), with their typical elongated and narrow monocot leaves, the area of individual leaves is linearly related to the product of length and maximum width (Stickler et al. 1961; Dwyer and Stewart 1986; Birch et al. 1998). For sorghum, each emerging leaf is longer and wider than the previous one, in most cases reaching a maximum around the third or fourth leaf below the flag leaf (Borrell et al. 2014a). Length and width of the largest leaf are indicative of total plant leaf area which, in turn, is associated with the water demand of plants via canopy transpiration (Stickler et al. 1961; Duncan et al. 1967; Borrell et al. 2014b; Kholová et al. 2014; George-Jaeggli et al. 2017). At the whole-plant level, leaf area index is commonly used as a critical variable when simulating photosynthesis- and/or transpiration-related crop productivity in cereals (Duncan et al. 1967; Wu et al. 2018; Chenu et al. 2018; Hammer et al. 2019). In combination with leaf angle, the size of the largest leaf is also a key component of canopy structure and influences light capture and canopy photosynthesis (Duncan 1971; Zhi et al. 2022a; Birch et al. 1998).

At the leaf level, leaf size is correlated with many physiological traits and their interplay can affect functional aspects of leaf gas exchange (Parkhurst and Loucks 1972; Chatterjee et al. 2016; Cano et al. 2019; Pan et al. 2021). As a component of leaf size, leaf width is closely associated with variation in leaf structural properties (e.g. vein density or patterning), which impacts the access of CO_2_ to chloroplasts and also water transport efficiency (Evans 1999; Giuliani et al. 2013; Baird et al. 2021). Similarly, leaf width in C_4_ crops has recently been found to be positively correlated with stomatal conductance, while being negatively associated with both mesophyll conductance and intrinsic water use efficiency (*i*WUE, defined as the ratio of CO_2_ assimilation rate to stomatal conductance at the leaf segment level) (Cano et al. 2019; Pan et al. 2021). This could be attributed to the negative association between leaf width and vein density, with the latter usually being positively correlated with stomatal density (Fiorin et al. 2016; Baird et al. 2021). Greater interveinal distance (smaller vein density) observed in wider leaves (Baird et al. 2021) results in reduced mesophyll conductance and a greater requirement for increased stomatal conductance to CO_2_ (greater stomatal opening) to maintain high photosynthetic rates (Pan et al. 2021), which at the same time leads to an increase in transpiration, and hence decreased *i*WUE (Crookston and Moss 1974; Farquhar and Sharkey 1982; Evans et al. 2009). Wider leaves also have a greater boundary layer resistance, which influences vapour transfer and heat exchange, and induces increased stomatal conductance (Parkhurst and Loucks 1972), which further contributes to reduced *i*WUE (Baird et al. 2021). A recent detailed study into the effects of leaf width on gas exchange, boundary-layer effects and leaf anatomy using 48 diverse field-grown genotypes in sorghum, corroborated these relationships between leaf width and *i*WUE (Pan et al. 2021). Therefore, leaf width may represent a potential breeding target to improve adaptation of sorghum to the hotter and drier climates of the future (Haussmann et al. 2012; Menamo et al. 2021), particularly if the leaf-level effects of leaf width on *i*WUE can scale up to transpiration efficiency (TE) at the plant level. Whole-plant TE has previously been identified as an important target trait for advancing adaptation relevant to future climates (Hammer et al. 2020), and useful genetic variation has been reported in sorghum (Hammer et al. 1997; Geetika et al. 2019; van Oosterom et al. 2021).

In order to efficiently select for optimal leaf width in a plant breeding program, it is important to understand the genetic basis of the trait and its interactions with other traits. The genetic control of leaf width has been investigated in rice (*Oryza sativa*) (Li et al. 1998; Yue et al. 2006; Zhao et al. 2011; Wang et al. 2012; Tang et al. 2018; Wen et al. 2020) and maize (Tian et al. 2011; Ku et al. 2012; Yang et al. 2016; Zhao et al. 2018; Fu et al. 2019), demonstrating that leaf width is under complex genetic control. For example, a comprehensive maize genome-wide association study (GWAS) using a Nested Association Mapping population (*n* = 4892) detected 295 significant single nucleotide polymorphisms (SNPs) associated with variation in leaf width, suggesting that leaf width is controlled by multiple QTL, most with effect sizes of less than 3 mm (Tian et al. 2011). In a recent mapping study in rice using a chromosomal segment substitution line population, nine leaf width QTL explaining 55.8% of phenotypic variation were detected (Tang et al. 2018). In addition, a *narrow leaf* gene (*Nal1* in rice and *ns1* in maize) was identified, which decreased leaf width in maize (Nardmann et al. 2004) and was associated with increased photosynthetic capacity in rice (Tang et al. 2018). The changes in leaf width of *Nal1* mutants were attributed to changes in the number of longitudinal leaf veins in rice (Qi et al. 2008; Takai et al. 2013), indicating a close linkage between leaf width and leaf vein or stomatal features as reported recently (Pan et al. 2021). Despite a number of genetic mapping studies in other cereals and the growing evidence that leaf width and stomatal features are tightly linked (Pan et al. 2021), work in sorghum has previously been limited to a few studies (Feltus et al. 2006; Sakhi et al. 2013; Kapanigowda et al. 2014; Shehzad and Okuno 2015; McCormick 2017). These previous sorghum studies have typically been of small population size (ranging from 107 to 370 individuals) and only detected eleven leaf-width QTL. Therefore, a more comprehensive study was needed to dissect the genetic basis of leaf width in sorghum.

Sorghum, the world’s fifth most important cereal, is morphologically and genetically diverse (Reddy and Patil 2015; Tao et al. 2021a) and can hence be adapted to a wide range of environments. Cultivated sorghum can be classified into five basic races, including *guinea*, *caudatum*, *kafir* and *durra*, which are traditionally grown in different agro-ecological zones (Harlan and Wet 1972). Evaluating differences in leaf width among the sorghum races might provide information about the importance of the trait for environmental adaptation (Lasky et al. 2015; Menamo et al. 2021) and hence its potential in breeding sorghum varieties with improved adaptation to variable climates (Hammer et al. 2020).

The objectives of the present study were to (i) investigate whether leaf width affects plant-level TE using lysimetry experiments; (ii) quantify the natural variation in leaf width using a sorghum diversity panel; (iii) examine whether there are differences in leaf width among sorghum races to understand its role in adaptation to different environments; and (iv) detect the genetic basis of leaf width using GWAS and identify potential candidate genes.

## Materials and methods

### Experimental design and genotypes used in lysimetry experiments

Two experiments (Exp1 and Exp2) were conducted using a fully automated lysimetry platform inside a semi-controlled enclosure at the University of Queensland, Gatton Campus (27°33´ S, 152°20´ E) in southeast Queensland, Australia. The automated lysimetry platform has been described previously (Chenu et al. 2018). Briefly, the enclosure has a solar weave roof and meshed poly weave sides, plus a gable fan that provides additional airflow when air temperature inside the enclosure exceeds 38 °C. Temperature and humidity inside the enclosure are automatically recorded every ten minutes (Table S1). Single plants were grown in large pots (at least 50 L capacity) to provide non-stressed growing conditions for sorghum. The amount of water used per plant was monitored every ten minutes by automatic weighing of the pots, which were set up on individual load cells mounted on trolleys. Each trolley held two rows of four pots and trolleys were setup such that the overall experiments had six rows and six columns. Individual pots were watered automatically via a PVC access tube embedded in the soil. In both experiments, pots were rewatered to their initial weight as soon as they dropped below a threshold value of 65 kg, at which time the soil moisture content was at approximately 50% of its drained upper limit.

Six diverse grain sorghum genotypes, which were known to cover a range of stomatal conductance and leaf widths (unpublished data), were grown in Exp1. In Exp2, twelve genotypes were grown with known differences in C_4_ photosynthetic parameters determined via fitting CO_2_ response curves (Zhi et al. 2022b), in addition to known variability in leaf width; two genotypes were in common across both experiments (Table S2). Genotypes were assigned to lysimetry pots using a completely randomised block design with six (Exp1) and three (Exp2) replicates. Exp1 was sown on 29 October 2018 and harvested on 9 January 2019 (anthesis) and Exp2 was sown on 25 March 2019 and harvested on 13 May 2019 (prior to anthesis). Before sowing, each pot was lined with a plastic bag to facilitate removal of soil at harvesting. Additionally, a 30-cm PVC tube with a volume of 750 ml was embedded vertically into the soil for watering. Pots were filled with air-dried black vertisol soil from a field near Dalby, a sorghum-growing area in southeast Queensland. For each pot, 20 g of Scotts Osmocote Plus controlled release fertiliser (16%N, 3.5%P, 10%K) (Scotts Australia, Baulkham Hills, NSW, Australia) and 10 g of Dolomite were applied to the top thirty centimetres of soil. Five seeds were sown per lysimeter, and seedlings were gradually thinned until one seedling per pot was left once the plants were well established (17 DAS in Exp1 and 14 DAS in Exp2). At that time, the soil surface of each pot was completely sealed using plastic sheets, leaving only a small opening for the plants, thereby minimising water losses from soil evaporation. One teaspoon of Thrive (Yates Australia, Padstow, NSW, Australia) water soluble fertiliser (25%N, 5%P, 8.8%K) was added fortnightly to the watering tubes to ensure plants were adequately fertilised.

### Leaf width and plant water use data collection in lysimetry experiments

The number of fully expanded and senesced leaves on mainstems and tillers were recorded weekly. A leaf was considered fully expanded when its ligule was visible above the ligule of the previous leaf and considered senesced once less than 50% of its area was green. Once fully expanded, each leaf was measured to determine its width (measured across the widest part of the leaf). Water use data was aggregated hourly based on the weight differences of each pot. Water use per plant for the entire experimental period was determined by calculating overall water use of each pot from the time of being sealed until harvest date, including the fresh weight of each plant. At harvest, plants were cut at the base of the stem and dried in a fan-forced dehydrator at 80 °C until a stable dry weight was reached. Plant TE (g kg^−1^) was taken as final dry mass divided by total water use of each plant.

### Experimental design and plant material used in field trials

To explore the genetic basis of leaf width, 701 lines from a sorghum diversity panel were used. The diversity panel has been described previously (Tao et al. 2020) and consists primarily of lines from the Sorghum Conversion Program, in which genes for standard height and maturity have been introgressed into diverse sorghum lines and landraces from all over the world (Rosenow et al. 1997). Three field experiments were conducted during three consecutive summer cropping seasons at Hermitage Research Facility (HRF), Warwick, Queensland, Australia (28°12’S, 152°5’E, 470 m above sea level), and Gatton Research Station (GAT), Gatton, Queensland, Australia (27°33’S, 152°20’E, 94 m above sea level). At HRF, two experiments (HRF1 and HRF2) were sown on 6 December 2016 and 8 January 2018, respectively. The third experiment was sown on 14 January 2019 at GAT. In all experiments, genotypes were assigned to plots using randomised row-column designs with partial replications (around 30% of genotypes being replicated). Each plot planted to one genotype comprised four rows and was 3 m wide and 4.5 m long. Experiments were planted with a target population density of 50,000 plants ha^−1^ at HRF and 108,000 plants ha^−1^ at GAT. Experimental areas were fertilised sufficiently to provide nutrient non-limiting conditions. HRF1 and HRF2 were sown on full sub-soil moisture profiles and only irrigated at sowing to enable establishment, whereas GAT was irrigated regularly to minimise the risk of water limitation during summer.

### Phenotyping and genotyping in field trials

Around two weeks after flowering, one plant per plot was randomly chosen from the middle two rows to measure leaf width. Measurements were made on the largest mainstem leaf, as the largest leaf has been found to be most closely correlated with total plant leaf area in maize plants (Stickler et al. 1961; Birch et al. 1998). The largest leaf in the genotypes from the diversity panel was found to be generally the third or fourth leaf below the flag leaf (Borrell et al. 2014a), however, the final number of leaves varies depending on maturity of each genotype. Width of the largest leaf was therefore found to be affected by final leaf number, which in turn is largely driven by flowering time in sorghum (Hesketh et al. 1969). Both traits were also measured; the date when more than 50% of the plants in a plot reached anthesis was recorded as days to flower for that plot. Final leaf number was determined by consecutively numbering leaves with a permanent marker as they appeared on one tagged plant per plot. To minimise the influence of flowering time, leaf width of the largest leaf was adjusted for flowering time. Additionally, the association between days to flower and final leaf number for 625 lines was evaluated in HRF2.

All 701 diversity panel entries were genotyped by Diversity Arrays Technology Pty Ltd (https://www.diversityarrays.com/technology-and-resources/dartreseq/). DNA was extracted from young leaf tissue of five plants in each plot using a modified cetyl trimethyl ammonium bromide (CTAB) method (Doyle 1987). The samples were digested with methylation-sensitive restriction enzymes (HpaII, MseI) to remove repetitive sequences prior to sequencing of the most informative representations of genomic DNA on next-generation sequencing platforms (Illumina, HiSeq 2500). The sequence data generated was aligned to version v3.1 of the sorghum reference genome sequence (McCormick et al. 2018) to identify SNPs (Single Nucleotide Polymorphisms).

### Statistical analyses

#### Phenotypic data

All statistical analyses were conducted using the R open-source statistical programming language (Team 2018). The relationship between plant TE and leaf width was evaluated by linear regression in Exp1 and Exp2 (Table S2). Averaged leaf width of leaf 10–12 in Exp1 and that of leaf 7–9 in Exp2 were used to determine the association with plant TE to avoid the confounding effect of total leaf number on the genotypic differences in leaf width, given these leaves were in the middle of increasing leaf size and active vegetation growth.

In the field trials, best linear unbiased predictors (BLUPs) of width of the largest leaf were calculated to minimise environmental effects within and across experiments using a restricted maximum likelihood (REML) by fitting a linear mixed model using the ASReml-R package (equation [1]) (Gilmour et al. 1997; Butler et al. 2018). To remove the variation in leaf width of the largest leaf caused by differences in days to flower which is closely associated with final leaf number, leaf width of the largest leaf adjusted for days to flower of each line was predicted by including days to flower as a fixed effect within each trial in the model [1]. Data from all trials was appended to allow for a multi-environment analysis using the following model.

1 $$y = X\beta + Zu + \varepsilon$$

where the response vector ***y*** is modelled by all the fixed effects ***β***, random effects ***u*** and all the residual effects ***ε***. The matrix ***X*** represents the design matrix for the fixed effects, and the matrix ***Z*** is the design matrix for the random effects. The fixed effects were composed of main effects for every trial plus any effects associated with linear changes along the rows and columns for every trial. The random effects contained sources of error within each trial including replication and any trial specific random row and column effects. The residual effects included trial specific residuals effects and first order auto-regressive (AR1) effects in both the row and column directions for each trial. This model included genotype as a random effect to predict genotype BLUPs within trials. The G × E was included in the model with an experiment by genotype interaction with a correlated variance structure with a separate genetic variance for each trial and pairwise correlations between each pair of trials. All possible sources of variation in the BLUPs were allowed for in the linear mixed model (Gilmour et al. 1997). A generalised measure of heritability was calculated due to the complex variance structure of the fitted model. The calculation of generalised heritability for each trial is given by equation [2].

2 $$H^{2} = 1 - \overline{{{\text{SED}}}}^{2} /\left( {2\sigma_{g}^{2} } \right)$$

where ${\varvec{H}}^{2}$ is the generalised heritability, ${\varvec{\sigma}}_{{\varvec{g}}}^{2}$ represents the genetic variance and $\overline{{{\text{SED}}}}$ is the average standard error of difference (Cullis et al. 2006).

The 701 genotypes in the diversity panel were assigned to racial groups, based on population structure results reported previously (Tao et al. 2020). However, the cut-off of genetic identity to select racial representatives (374 genotypes in total) was adjusted to 70% within a racial group for valid inter-group comparisons. The racial differences in leaf width were analysed independently for HRF1, HRF2 and GAT, using Wald tests.

#### Genome-wide association studies and identification of QTL and candidate genes for leaf width

The GWAS analyses were performed using the software FarmCPU (Liu et al. 2016). A Bonferroni-corrected significant threshold was calculated based on 0.05/number of effective tests (Duggal et al. 2008; Li et al. 2012, 2021). The significant SNPs detected across locations were further clustered into unique QTL based on a 1-cM window, in accordance with the linkage disequilibrium decay previously identified in the diversity panel (Tao et al. 2020), 200 kb, which is equivalent to ~ 1 cM (Mace and Jordan 2011).

Leaf width QTL previously identified in sorghum (Feltus et al. 2006; Sakhi et al. 2013; Kapanigowda et al. 2014; Shehzad and Okuno 2015; McCormick 2017) were compared with the QTL identified in the current study, using the predicted locations of the QTL CI from the Sorghum QTL Atlas (Mace et al. 2019). In addition, inter-species QTL comparisons for leaf width were conducted, specifically in maize (Tian et al. 2011) and rice (Tang et al. 2018), where the maize and rice QTL were projected onto the sorghum consensus map following the same methodology as described previously (Mace et al. 2019). Thirty-two sorghum orthologues of a priori candidate genes (Table S3), compiled previously (Tian et al. 2011), were used to evaluate the proximity of the QTL detected in the sorghum diversity panel based on 1 cM window. To further validate the function of a priori candidate genes, haplotype analysis (Minor allele frequency > 5%) was conducted in R using packages “ape” (Paradis et al. 2004) and “pegas” (Paradis 2010), as previously described (Tao et al. 2021b).

## Results

### Correlation between plant water use efficiency (TE) and leaf width in the lysimetry experiments

**Fig. 1:**
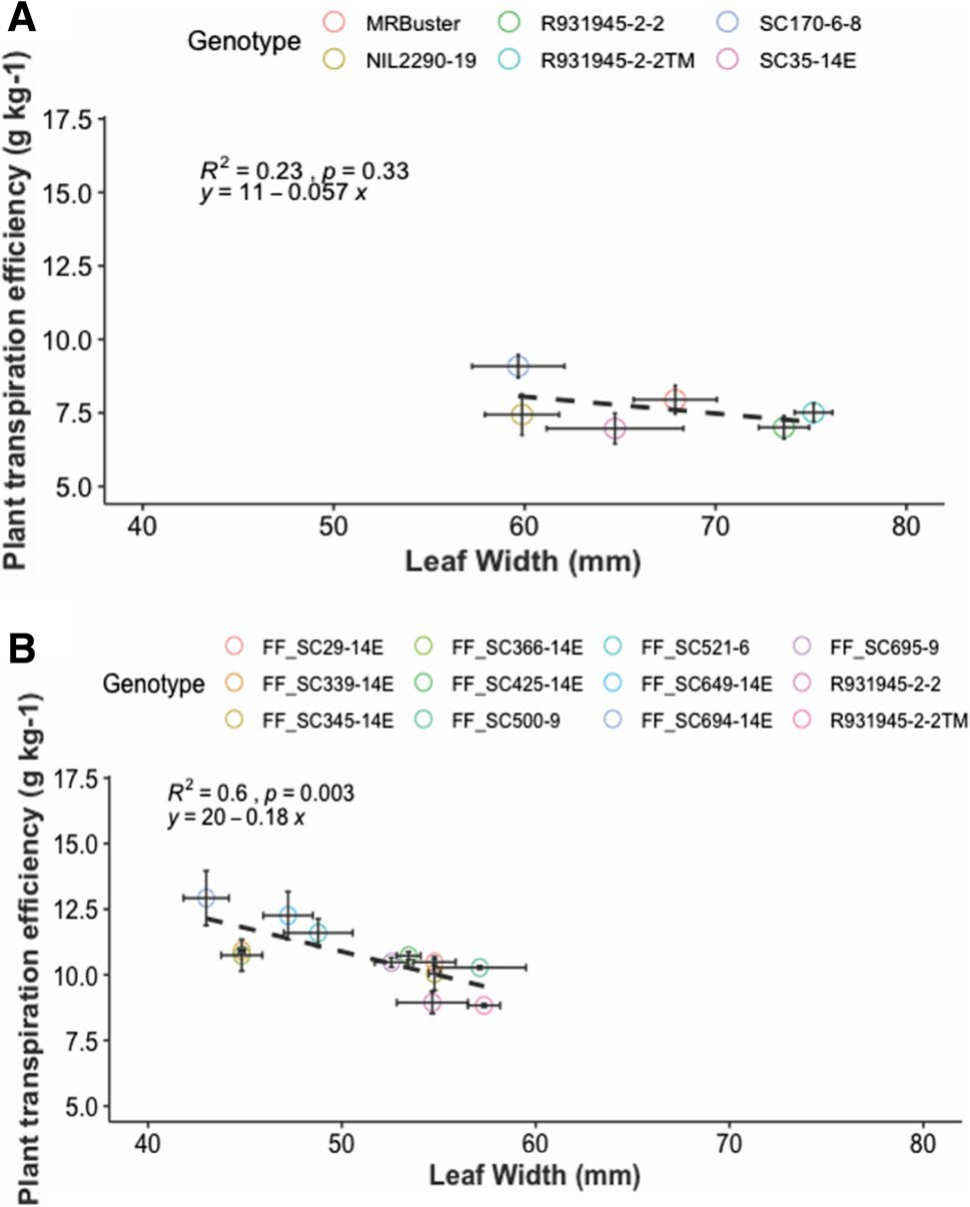
Relationship between plant transpiration efficiency and leaf width across genotypes in Exp1 (**A**) and Exp2 (**B**) Note: leaf width: the mean of leaves 10–12 in Exp1 (**A**) and leaves 7–9 in Exp2 (**B**); *R*^*2*^: coefficient of determination of the relationship; *p*: significance level; error bars on each data point indicate standard errors of plant transpiration efficiency and leaf width

In the semi-controlled lysimetry experiments, a negative correlation between plant TE and average leaf width (across leaves 10–12 and leaves 7–9 in Exp1 and Exp2, respectively) was observed in both Exp1 and Exp2 (Fig. 1). This correlation was particularly strong and significant among the twelve genotypes in Exp2 (Fig. 1B: *R*^2^ = 0.6, *p* = 0.003). The correlation was not significant across the six genotypes in Exp1 (Fig. 1A: *R*^2^ = 0.23, *p* = 0.33); however, a consistent trend was shown in the regression of plant TE versus leaf width.

### Association of leaf width with days to flower in the sorghum diversity panel

**Fig. 2:**
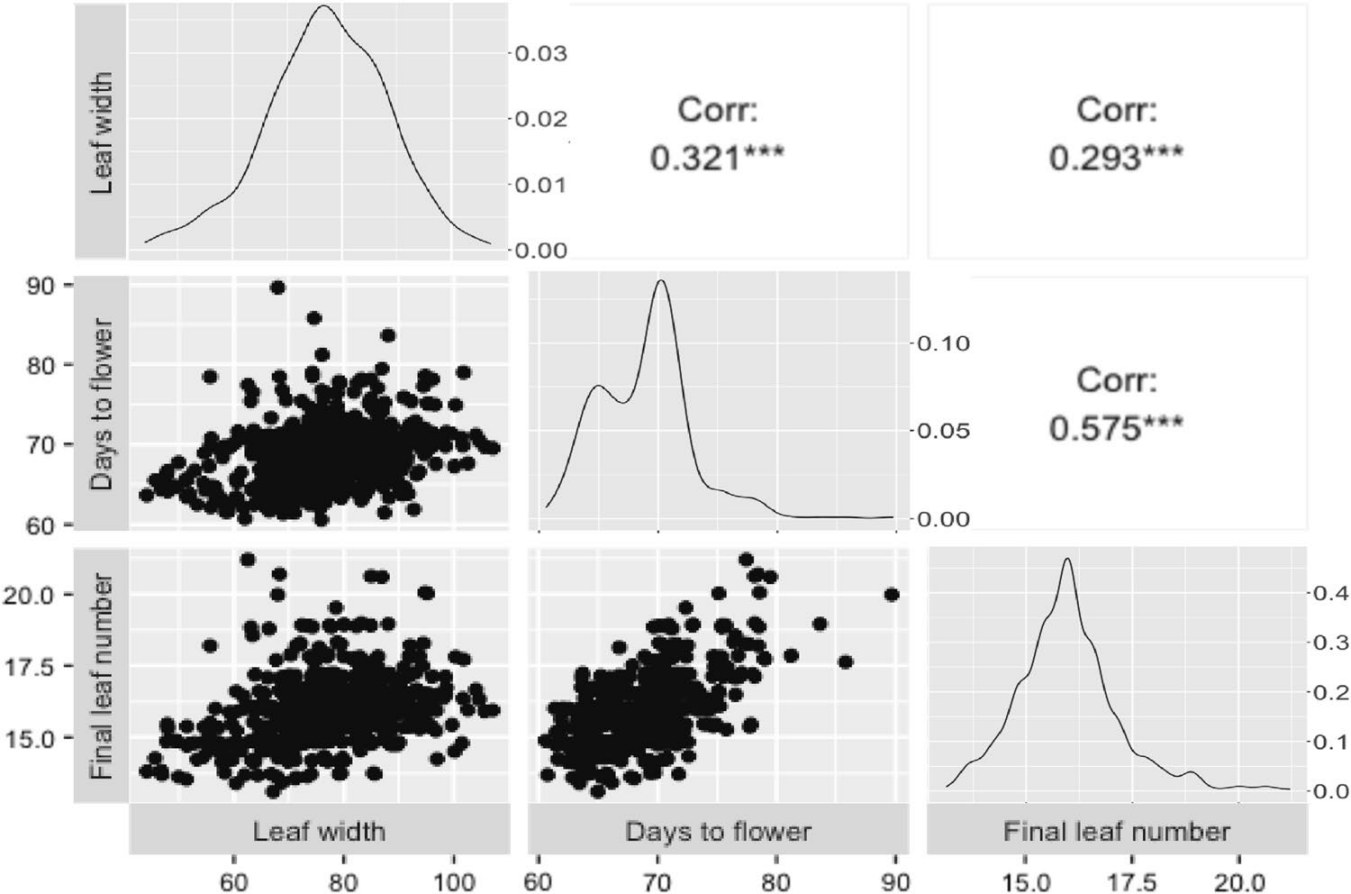
Correlation among leaf width of the largest leaf within a plant (mm), days to flower and final leaf number in HRF2 (*n* = 625 lines) Note: Data shown is leaf width, days to flower and final leaf number BLUPs. Leaf width in mm is shown on the x-axes of all three panels on the left, days to flower on the panels in the middle and final leaf number on the panels on the right. Final leaf number is shown on the y-axes of all panels in the bottom row, days to flower on the panels in the middle and leaf width in mm on all panels in the top row. The variable names are displayed on the outer edges of the matrix. The boxes along the diagonals display the density plot for each variable. The boxes in the lower left corner display the scatterplot between each variable. The boxes in the upper right corner display the Pearson correlation coefficient between each variable plus significance levels as stars (***, **, * correspond to *p* < 0.001, 0.01, 0.05, respectively). Axis labels are for the bottom and left panels

To evaluate the effect of flowering time on width of the largest leaf, correlation analysis was conducted among leaf width, days to flower and final leaf number in HRF2 (*n* = 625 lines). Days to flower and final leaf number were highly correlated with each other as expected (*r* = 0.58). Significant correlations of width of the largest leaf with days to flower (*r* = 0.32) and final leaf number (*r* = 0.29) were also found, suggesting that about 10% of variation in width of the largest leaf could be driven by differences in flowering time (Fig. 2). Therefore, to minimise the confounding effects of differences in flowering time on width of the largest leaf, GWAS analyses were performed based on BLUPs of leaf width adjusted for days to flower.

### Phenotypic variation in leaf width in the sorghum diversity panel

Leaf width varied from 43.1 to 116.8 mm across three trials (Table 1). Strong correlations (~ 0.80) among trials and high heritability (on average 0.85) were observed, suggesting high levels of genetic effects on leaf width across trials with low levels of interactions between genotypes and environments (G × E) (Table 1). This suggests that a large proportion of leaf width variation is attributed to genetic differences among sorghum lines in the diversity panel (Table 1).

**Table 1:** Correlation among the field trials and summary of minimum, maximum, mean, standard error and heritability for leaf width across the three field trials

			HRF1	HRF2	GAT
Leaf width	Correlations	HRF1	1	0.79	0.81
		HRF2	0.79	1	0.78
		GAT	0.81	0.78	1
	Summary	Min	55.3	44.4	43.1
		Max	114.6	103.7	116.8
		Mean	85.2	74.6	82.5
		std.error	5.4	6.1	6.3
		H^2^	0.83	0.85	0.86

Data shown is leaf width of the largest leaf adjusted for days to flower BLUPs; HRF2: diversity panel grown at Hermitage Research Facility in Warwick QLD in 2018; different letters mean statistically significant differences in leaf width determined through pairwise comparison among the five racial groups within each trial

### Differences in leaf width among sorghum races in the diversity panel

**Fig. 3:**
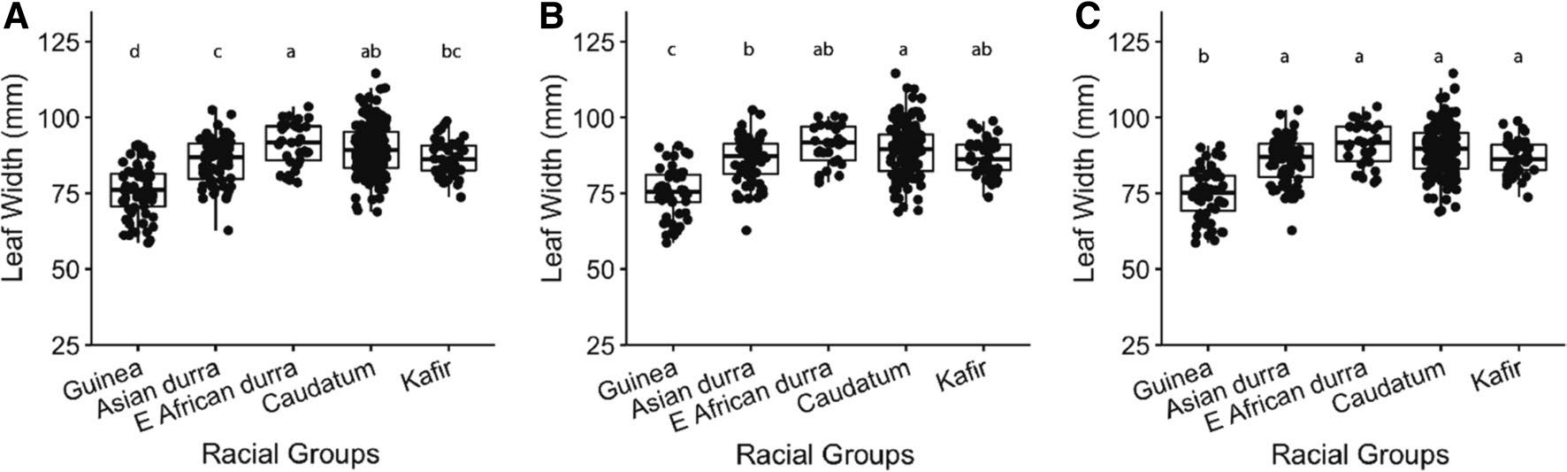
Leaf width for genotypes assigned to five sorghum racial groups in the trials of HRF1 (**A**), HRF2 (**B**) and GAT (**C**) Note: Data shown is leaf width BLUPs of the largest leaf adjusted for days to flower; Min: the minimum leaf width; Max: the maximum leaf width; Mean: the mean leaf width; std.error: standard error; H^2^: generalised heritability; HRF1: diversity panel grown at Hermitage Research Facility in Warwick QLD in 2017; HRF2: the diversity panel grown at Hermitage Research Facility in Warwick QLD in 2018; GAT: the diversity panel grown at Gatton Research Facility in Gatton, QLD in 2019

Lines from the diversity panel used in these trials were assigned to five racial groups. Significant differences in leaf width among these groups were observed and groups ranked consistently across the three field trials (Fig. 3, *p* < 0.001, Wald tests). Racial groups explained ~ 70% of the observed variation in leaf width. The *guinea* racial types exhibited significantly narrower leaves compared with the remaining four racial groups in all trials, while the East African *durra* types showed wider leaves than the other racial types (Fig. 3).

### GWAS for leaf width in the diversity panel

**Fig. 4:**
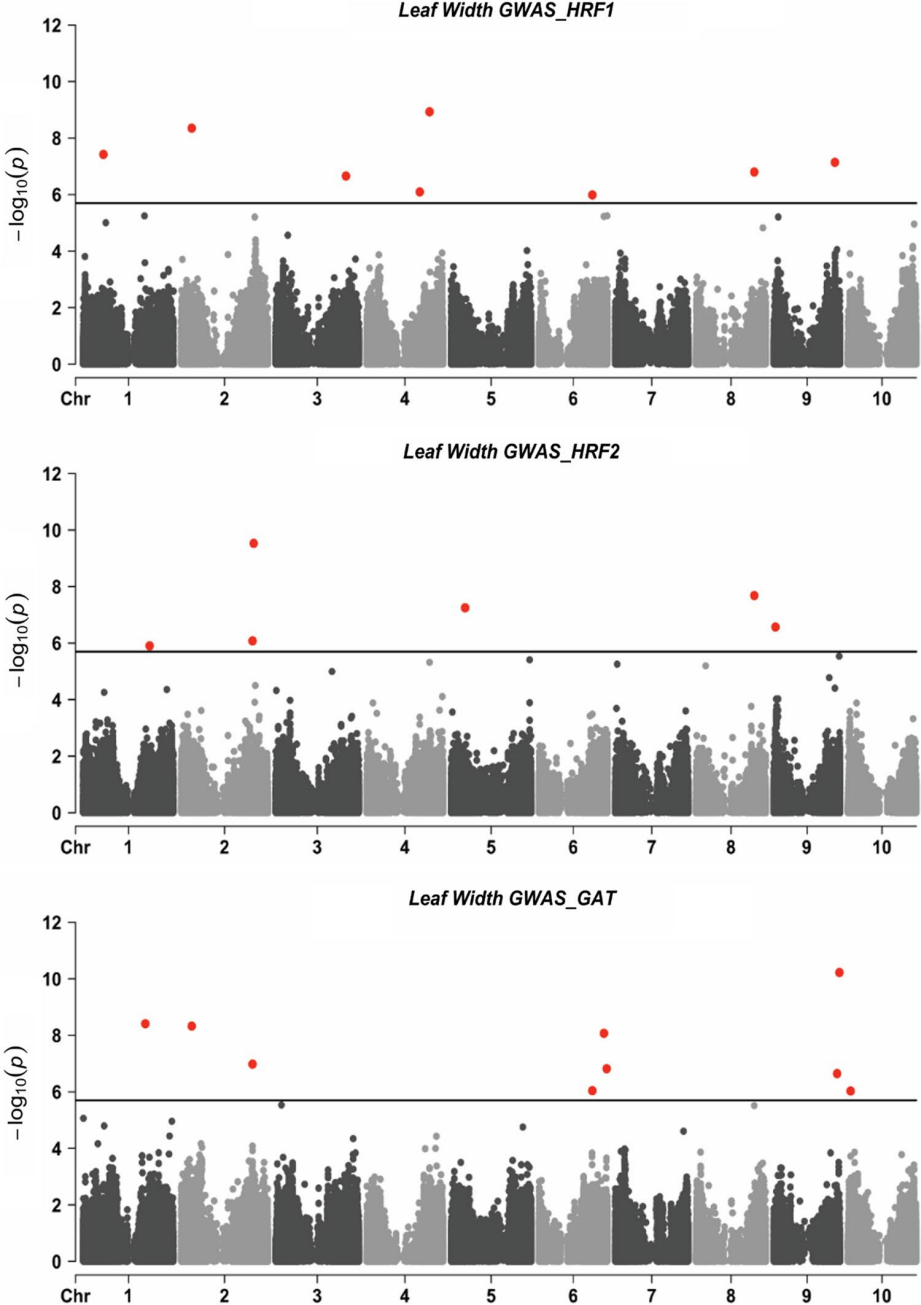
Manhattan plot of leaf width in HRF1, HRF2 and GAT Note: HRF1: diversity panel grown at Hermitage Research Facility in Warwick QLD in 2017; HRF2: diversity panel grown at Hermitage Research Facility in Warwick QLD in 2018; GAT: diversity panel grown at Gatton Research Facility in Gatton, QLD in 2019; the SNPs in red are significant ones detected using the *p*-value < 2e-6

In total, 414,899 SNPs were identified in the diversity panel and genetic (centimorgan, cM) positions of the SNPs were predicted using a sorghum consensus map (Mace et al. 2019). The GWAS analyses were performed with 354,715 SNPs with minor allele frequency (MAF) > 0.01 and a Bonferroni-corrected significant threshold of *p* < 2e-6. Using BLUPs for leaf width of the largest leaf adjusted for days to flower in HRF1, HRF2 and GAT, 20 significant SNPs were detected (Fig. 4), resulting in 19 leaf width QTL based on a 1-cM window (Table 2 and Table S4). The leaf width QTL were distributed across all ten chromosomes in sorghum. Eleven leaf width QTL have been identified in sorghum from five previous studies (Feltus et al. 2006; Sakhi et al. 2013; Kapanigowda et al. 2014; Shehzad and Okuno 2015; McCormick 2017), which were conducted in relatively small populations incorporating 107 to 370 individuals. Based on a 1-cM overlap window (Table 2 and Table S4), three (16%) QTL in the diversity panel were found to be co-located with leaf width QTL identified previously, representing a significant enrichment (*p* < 0.05, Chi-square test). The remaining 16 were firstly reported in this study, representing additional genomic regions controlling leaf width that are available for further studies.

**Table 2:** QTL of leaf width detected in the diversity panel across three field trials

QTL	Chr	bp	cM	*p*-value	No.SNPs	MAF	Overlap_Sb	Dis_Sb (bp)	Dis_Zm (bp)	Dis_Os (bp)
qLW_dtf1.1	1	18,190,836	44.4	3.79E − 08	1	0.12	N	39,822,295	63,291	NA
qLW_dtf1.2	1	55,679,813	64.4	3.90E − 09	1	0.40	Feltus et al. (2006)	2,333,318	969,278	NA
qLW_dtf1.3	1	59,543,465	76.0	1.25E − 06	1	0.11	N	1,508,873	196,747	NA
qLW_dtf2.1	2	9,445,603	47.0	4.48E − 09	2	0.12	Feltus et al. (2006)	305,326	283,480	197,821
qLW_dtf2.2	2	63,840,929	143.1	1.05E − 07	2	0.44	Feltus et al. (2006)	798,815	280,653	54,197,505
qLW_dtf2.3	2	64,864,314	145.1	2.97E − 10	1	0.06	N	1,822,200	336,741	55,220,890
qLW_dtf3.1	3	62,800,310	123.6	2.22E − 07	1	0.04	N	3,663,292	128,428	NA
qLW_dtf4.1	4	47,570,871	74.8	8.08E − 07	1	0.28	N	15,410,360	2,541,923	29,204,706
qLW_dtf4.2	4	56,350,841	103.8	1.18E − 09	1	0.07	N	6,630,390	760,752	37,984,676
qLW_dtf5.1	5	12,639,592	62.3	5.65E − 08	1	0.08	N	9,133,817	171,741	10,981,001
qLW_dtf6.1	6	47,762,800	83.1	9.12E − 07	2	0.34	N	3,998,325	352,174	4,589,766
qLW_dtf6.2	6	58,131,040	156.5	8.51E − 09	1	0.36	N	6,366,040	581,867	1,276,768
qLW_dtf6.3	6	60,570,035	165.2	1.52E − 07	1	0.10	N	8,805,035	49,460	1,162,227
qLW_dtf8.1	8	52,131,634	67.1	2.08E + 08	2	0.21	N	5,226,366	3,021,276	NA
qLW_dtf9.1	9	1,543,090	20.5	2.70E − 07	1	0.25	N	1,049,016	310,434	NA
qLW_dtf9.2	9	54,737,199	112.2	7.25E − 08	1	0.17	N	2,834,733	103,710	NA
qLW_dtf9.3	9	56,705,097	115.7	2.27E − 07	1	0.10	N	4,802,631	14,574	NA
qLW_dtf9.4	9	58,789,384	133.9	6.01E − 11	1	0.05	N	6,886,918	16,831	NA
qLW_dtf10.1	10	2,537,235	29.1	9.35E − 07	1	0.29	N	NA	762,168	53,479,135

“Chr”: chromosome; “bp”: the physical locations based on sorghum genome v3.1; “cM”: the genetic linkage positions based on the sorghum consensus map; “*p*-value”: the minimum p-value of SNPs in the QTL; “No.SNPs”: number of significant SNPs included in a QTL; “MAF”: minor allele frequency; “Overlap_Sb” the overlapping of leaf width the sorghum diversity panel with previously identified leaf width QTL in sorghum (Feltus et al. 2006; Sakhi et al. 2013; Kapanigowda et al. 2014; Shehzad and Okuno 2015; McCormick 2017), “N” shows no overlapping based on 1 cM window; “Dis_Sb (bp)”: the distance (bp) of leaf width QTL in the sorghum diversity panel from their closest leaf width QTL in previous sorghum studies (Feltus et al. 2006; Sakhi et al. 2013; Kapanigowda et al. 2014; Shehzad and Okuno 2015; McCormick 2017); “Dis_Zm (bp)”: the distance (bp) of QTL detected in the sorghum diversity panel from their closest leaf width QTL identified in the maize-NAM study (Tian et al. 2011), via projecting maize QTL onto the sorghum consensus map; “Dis_Os (bp)”: the distance (bp) of QTL detected in the sorghum diversity panel from their closest leaf width QTL identified in the rice study (Tang et al. 2018), via projecting rice QTL onto the sorghum consensus map; “NA”: no leaf width QTL was identified on this chromosome in previous studies

In addition, compared with projected locations of previously identified maize leaf width QTL (Tian et al. 2011) on the sorghum consensus map (Mace et al. 2019), eight (42%) sorghum QTL overlapped with maize QTL based on a 1 cM (Table 2 and Table S4). This represented a significant enrichment with maize leaf width QTL (*p* < 0.05, Chi-square test). Comparing the sorghum QTL for leaf width identified in this study with the nine leaf width QTL identified from a recent rice study (Tang et al. 2018), four (21%) were less than 5 cM from the projected locations of leaf width QTL in rice (Tang et al. 2018; Mace et al. 2019) (Table 2 and Table S4). Enrichment analysis found that the level of correspondence was significant (*p* < 0.05, Chi-square test), based on a 5-cM window, indicating similarities between the genetic bases of leaf width in sorghum and rice.

### A priori candidate genes for leaf width in the diversity panel

In total, three a priori candidate genes overlapped with leaf width QTL identified in this study based on a 1-cM threshold (Table 3). These three a priori candidate genes have been reported to be involved in leaf patterning either via cell specification and differentiation, such as abaxial or adaxial cell specification, or expansion and proliferation of cell groups that affect leaf architectural traits including leaf length and width (Nogueira et al. 2007; Strable et al. 2017; Zhong et al. 2021).

**Table 3:** A priori candidate genes and the closest leaf width QTL in the diversity panel

A priori candidate gene	Original gene ID	Species	Chr	bp	cM	QTL	Dis_bp	Dis_cM	Reference
*Sobic.001G199200*	*GRMZM2G167824*	Maize	1	18,036,971	44.3	qLW_dtf1.1	153,865	0.1	Strable et al. (2017)
*Sobic.003G298600*	*GRMZM5G874163*	Maize	3	62,996,199	124.5	qLW_dtf3.1	195,889	0.9	Zhong et al. (2021)
*Sobic.008G070600*	*GRMZM2G020187*	Maize	8	9,201,092	67.1	qLW_dtf8.1	42,930,542	0.0	Nogueira et al. (2007)

“Original gene”: the previous genes identified to be associated with leaf development; “Chr”: chromosome, “bp”: the mid-point of physical position in bp; “cM”: the genetic position on the consensus map; “QTL”: the closest QTL of leaf width in the diversity panel to the candidate gene; “Dis_bp”: the distance between the candidate genes and the closest QTL in bp; “Dis_cM”: the distance between the candidate genes and the closest QTL in cM

**Fig. 5:**
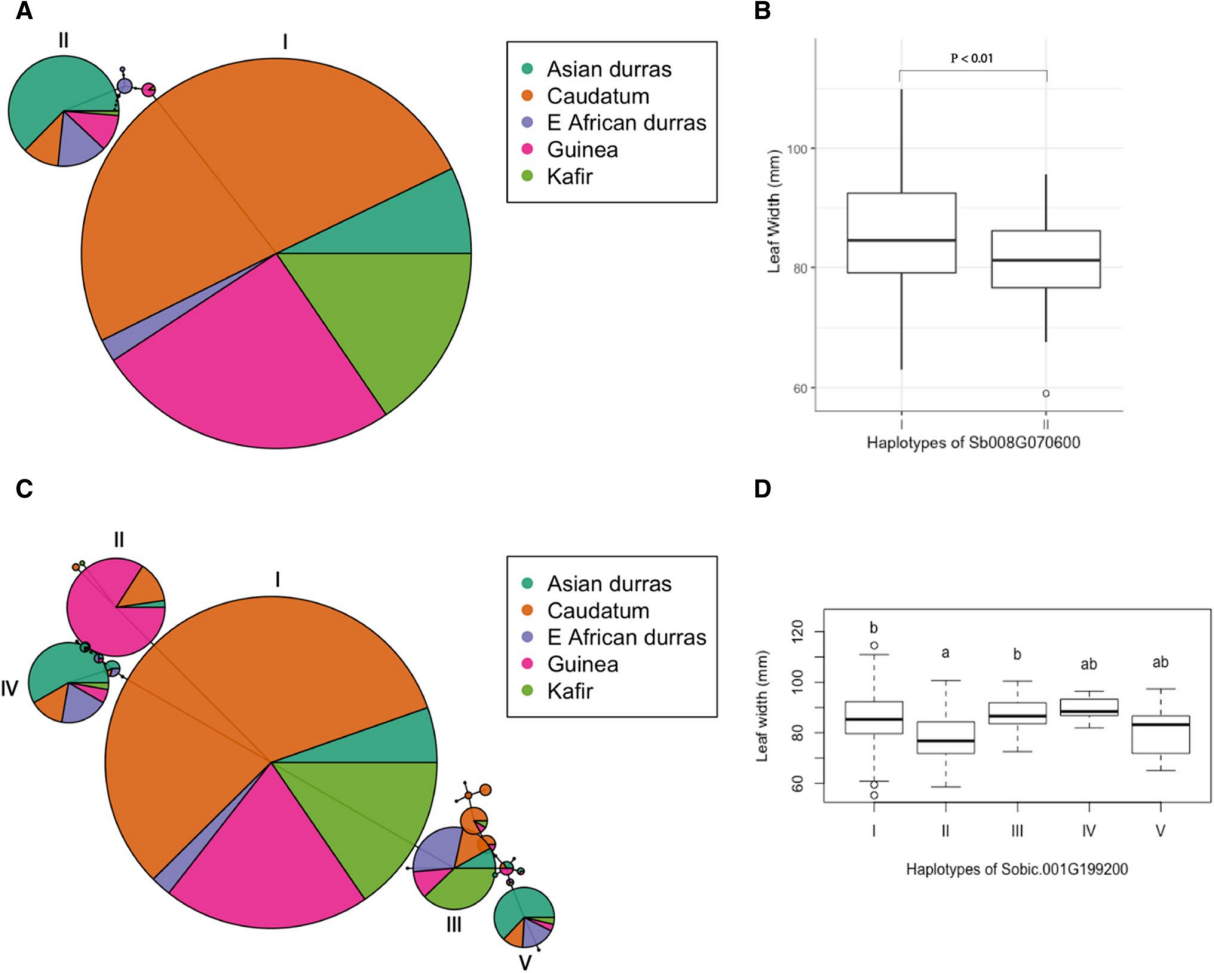
Haplotype network of *Sb008G070600* (**A**) and *Sb001G199200* (**C**), and boxplots showing effects of major haplotypes of *Sb008G070600* (**B**) and *Sb001G199200* (**D**) on leaf width Note: *P* value in plot B indicates the difference in leaf width between two haplotypes by t-test; different letters over the boxes in plot D mean statistically significant differences in leaf width determined through Tukey-pairwise comparison among the five major haplotypes of *Sb001G199200*

To further explore effect of the three candidate genes on leaf width, haplotype analysis was conducted. Two major haplotypes were identified in *Sobic.008G070600* (homolog of leafbladeless1 gene (*lbl*, *GRMZM2G020187*), co-located with qLW_dtf8.1 based on 0 cM), with haplotype I comprising mainly caudatum types and haplotype II comprising mainly Asian durra types (Fig. 5A). Significant difference in leaf width between these two major haplotypes was detected (Fig. 5B), consistent with the role of *Sobic.008G070600* in controlling leaf width. Moreover, a haplotype analysis for another a priori candidate genes, *Sobic.001G199200* (homolog of *yab11* (*GRMZM2G167824*) gene, 0.1 cM away from qLW_dtf1.1), showed five major haplotypes (Fig. 5C). Haplotype I mainly comprised caudatum types and haplotype II dominated in guinea types. Leaf width was significantly different among these five major haplotypes of *Sobic.001G199200* (Fig. 5 D). Regarding to *Sobic.003G298600* (0.9 cM away from qLW_dtf3.1), no SNPs were detected within the gene. In summary, the haplotype analyses for *Sobic.008G070600* and *Sobic.001G199200* further supported their strong association with leaf width in sorghum.

## Discussion

Leaf width is suggested to be an important component trait affecting plant-level TE in sorghum, as indicated by the negative association of plant-level TE with leaf width in both lysimetry experiments in this study. This has not been previously reported. The wide range of leaf widths observed in the diversity panel facilitated the dissection of the underpinning genetic control via GWAS. In addition, consistent and significant differences in leaf width among the five sorghum races suggested an association of leaf width with environmental adaptation. Across the three trials, 19 unique leaf width QTL were identified with high levels of overlap among leaf width QTL in sorghum, maize (Tian et al. 2011) and rice (Tang et al. 2018). This suggests likely similarities in the genetic basis of leaf width in cereals. Furthermore, three a priori candidate genes were confirmed based on a 1-cM threshold, which were characterized as regulating leaf patterning including dorsoventrality and expansion of the leaf blade.

### Leaf width was negatively associated with plant-level TE

Leaf width in C_4_ species has recently been found to be positively associated with stomatal conductance, while being negatively correlated with water use efficiency at the leaf level (*i*WUE) (Cano et al. 2019; Pan et al. 2021). However, it has not previously been reported whether this relationship scales up to the plant level in sorghum. In this study, a negative association between whole-plant TE and leaf width was shown in two lysimetry experiments, suggesting transpiration losses could potentially be reduced via decreased leaf width without trade-offs in biomass. While the association was moderately strong and significant in Exp2 which comprised twelve genotypes, the correlation between leaf width and plant TE was not significant in Exp1 (in which six genotypes were tested). However, the direction was consistent across both experiments. This is a significant finding, particularly since the negative relationship between water use efficiency and leaf width has only been investigated at the leaf level (Cano et al. 2019; Pan et al. 2021). Hence, dissecting the genetic basis of leaf width might be beneficial for improving water use efficiency in breeding, particularly, given the fact that small differences in plant-level TE are likely to have major effects on crop yield and adaptation (Hammer et al. 2020). However, a follow-up study is needed to further explore the extent of the relationship between leaf width and plant-level TE.

One of the underlying mechanisms associated with the negative relationship between water use efficiency and leaf width may be the strongly negative relationship between leaf width and the 1st and 2nd order vein density in C_4_ species (Baird et al. 2021). Narrow leaves are associated with high vein density and, in turn, with high density of bundle sheath cells, which increases the capacity for CO_2_ concentration and hence rates of photosynthetic assimilation (Dengler et al. 1994; Christin et al. 2013). Moreover, high vein density with small interveinal distance results in reduced stomata opening and thus lower stomatal conductance which, in turn, contributes to high *i*WUE (Pan et al. 2021). Apart from the low stomatal density, wide leaves are also associated with a thicker boundary layer, which reduces vapour flux from the leaf to the ambient air, thereby reducing stomatal resistance compared to narrower leaves with lower boundary layer resistance. The consequent slight increase in leaf temperature causes higher internal vapour pressure, which enhances transpiration. This reduces *i*WUE in the wider leaf type, especially at low wind speeds (Parkhurst and Loucks 1972). Narrow leaf types with thin boundary layers, which facilitate more direct vapour and heat exchange between leaves and the atmosphere, more easily maintain leaf temperature close to ambient air temperature while having higher stomatal resistance (Nobel 1999). Additionally, this association between leaf width and leaf temperature may be important to photosynthetic performance by preventing leaves from reaching damagingly high temperatures (Gates 1968; Parkhurst and Loucks 1972; Moore et al. 2021). In summary, the negative linkage between leaf width and water use efficiency (both *i*WUE and plant-level TE) could be attributed partly to its coordination with vein density and boundary layer, both of which are associated with stomatal features (e.g. density and opening) and thus with water use efficiency (Pan et al. 2021; Baird et al. 2021).

### Using leaf width in breeding to improve plant water use efficiency in different environments

The large natural variation, relative ease of measurement and high heritability of leaf width make it amenable to selection in breeding programs, as a potential surrogate for transpiration efficiency. However, the complex genetic basis underpinned by multiple small-effect QTL makes marker-assisted selection more difficult. A breeding approach using whole-genome information (e.g. genomic selection) could be an effective strategy (Fernandes et al. 2018). The complex genetic architecture and existence of many small-effect QTL also indicate that large populations are required to ensure sufficient power to identify the majority of QTL by GWAS. The fact that only eleven leaf width QTL have previously been detected is partly due to the small population sizes used (Feltus et al. 2006; Sakhi et al. 2013; Kapanigowda et al. 2014; Shehzad and Okuno 2015; McCormick 2017). The larger population used in the current study enabled detection of additional QTL and improved dissection of the genetic basis of leaf width in sorghum. The cross-species comparisons of leaf width QTL suggest conserved genetic control of leaf width across cereals. This highlights the important information that can be provided by using sorghum, which is genetically diverse and adapted to a range of environments, as a model for the genetic dissection of traits that will contribute to greater adaptation of cereals to future climates and a range of production systems.

Furthermore, the consistent differences in leaf width among sorghum races found in this study potentially reflects specific adaptation to their agro-ecological environments of origin (Morris et al. 2013; Menamo et al. 2021). However, given the interactions and linkages between leaf width, stomatal conductance, photosynthesis and water use efficiency, the extent to which there is any advantage in water use efficiency relative to leaf width will depend on the canopy and environment contexts, especially with regard to water availability (Tsukaya 2004). Under low resource availability, narrower leaves associated with higher vein density and thinner boundary layers, are expected to provide advantages in water use efficiency, as suggested in the present study. Narrow leaf types would also be advantageous in dry conditions because of reduced total plant leaf area, and therefore lower canopy transpiration over time, provided that narrow leaf width is not accompanied by a greater number of leaves or increased tiller number (Tardieu et al. 2018; Hammer et al. 2019). In addition to the reduced transpiration at both leaf and plant levels, narrow leaves might also be coordinated with leaf angle to improve radiation penetration throughout the canopy in water-stressed environments (Duncan 1971; Witkowski and Lamont 1991). In the absence of water limitation, the situation likely differs. At the leaf level, wider leaves with greater interveinal distance will have greater stomatal aperture, increased transpiration, and decreased water use efficiency (Baldocchi et al. 1985; Pan et al. 2021; Baird et al. 2021). However, at the canopy level, in such potential growth conditions, the greater stomatal conductance is conducive to enhanced photosynthesis and crop growth. A simulation analysis for sorghum quantified this trade-off and indicated a yield disadvantage of reduced plant-level TE in high-yielding environments (Hammer et al. 2019). Hence, the optimal leaf width is dependent on the environmental context.

### Regulating dorsoventrality may play a role in varying leaf width

The a priori candidate genes identified in the diversity panel, based on a 1-cM threshold, have previously been found to regulate dorsoventrality (Nogueira et al. 2007; Strable et al. 2017; Zhong et al. 2021). The dorsoventral axis is a prerequisite to lateral outgrowth of the leaf lamina thus playing an important role in establishing leaf blades (Waites and Hudson 1995), resulting in the adaxial surface for light capture and the abaxial surface for gas exchange (Tsukaya 2004; Yang and Hwa 2008). One of the a priori candidate genes identified here, *Sobic.008G070600* the orthologue of maize *leafbladeless1* (*lbl1, GRMZM2G020187*), has been shown to specify adaxial and abaxial organ polarity (Nogueira et al. 2007). The recessive mutations of *lbl1*, mainly expressed in the shoot apical meristem, vasculature and adaxially in leaf primordia, exhibited alteration in abaxialization and width of leaves (Nogueira et al. 2007). It has also been reported that *KANADI* and *YABBY* gene families regulate leaf width through controlling abaxialization (Candela et al. 2008; Ku et al. 2012). In the current study, *Sobic.001G199200*, orthologous to *GRMZM2G167824*, which is one of the *YABBY* family genes (Strable et al. 2017), was identified to be 0.1 cM away from qLW_dtf1.1. *GRMZM2G167824* has been shown to affect leaf width likely through regulating cell specification and differentiation during leaf development in maize (Strable et al. 2017). Moreover, *Sobic.003G298600*, the orthologue of *GRMZM5G874163*, has been reported to control leaf width and regulate the formation of distal tissues by coordinating with *KANAD1* family genes in Arabidopsis (*Arabidopsis thaliana*) as orthologue of *AT5G60450* (Pekker et al. 2005). In summary, processes associated with dorsoventrality are likely one of the molecular mechanisms underlying leaf width regulation in sorghum.

## Conclusions

The negative association between whole-plant TE and leaf width suggests that the effect of leaf width on *i*WUE at the leaf level could translate to the whole-plant level. The wide range and consistent differences in leaf width among the five sorghum races suggest that it might be associated with environmental adaptation. The QTL and candidate genes for leaf width identified in the diversity panel provide valuable information, facilitating modification of leaf width to improve plant TE and canopy characteristics to improve cereal adaptation to different agro-ecological environments and changing climates.

## Supplementary Information

Below is the link to the electronic supplementary material.

Supplementary file1 (XLSX 23 KB)

## Data Availability

The data used in the current study is available from the corresponding author upon request.
